# Evaluation of best management practices to reduce sediment yield in the upper Gilo watershed, Baro akobo basin, Ethiopia using SWAT

**DOI:** 10.1016/j.heliyon.2023.e20326

**Published:** 2023-09-21

**Authors:** Mengistu Zantet oybitet, Takele Sambeto Bibi, Eliyas Abdulkerim Adem

**Affiliations:** a Department of Hydraulic and Water Resources Engineering, Mizan Tepi University, Ethiopia; b Department of Water Supply and Environmental Engineering, Madda Walabu University, Ethiopia; c Department of Water Resources and Irrigation Engineering, Madda Walabu University, Ethiopia

**Keywords:** Best management practice, Sediment yield, SWAT, Upper gillo watershed

## Abstract

The increasing sediment yield in the watershed is caused by natural and human activities, which significantly shifts the hydro-meteorological in the watershed. The Modified Universal Soil Loss Equation (MUSLE) equation in the Soil and Water Assessment Tool (SWAT) was used to estimate sediment yields for each hydrological response unit (HRU) based on peak runoff, daily runoff volume, area of hydrological response unit, and other estimated and default hydrological model parameters. The amount of sediment yield from each HRU is then summed to give the total soil erosion for the watershed.The spatio-temporal variations of sediment yield in the Upper Gilo watershed was simulated to identify the hotspot area and select the effective management practices (BMPs) for reducing significant problems. Model calibration and validation were carried out using sediment yield data from 1990 to 2004 and 2005 to 2014. The results indicated that the watershed total sediment yield is 1021.8 tonnes/yr. Furthermore, 17 sub-basins (37.8% of total watershed area) are severely threatened by high soil erosion. According to the simulation results, the filter strips, terraces, and contours reduced the watershed sediment yield by up to 53.2%, 45.4%, and 48%. Overall, the selected BMPs are highly effective in reducing sediment yield in watershed-prone areas.

## Graphical abstract

**Figure:**
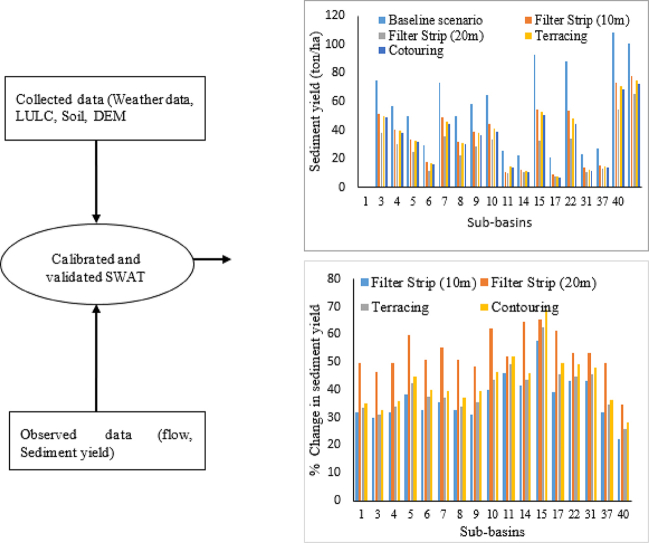

## 1 Introduction

The amount of erosive debris exported from watersheds is increasing due to human activity. Unsuitable land-use land cover activities, land degradation, and poor soil and water conservation practices in the upstream watershed have exacerbated the downstream sediment load [1,2]. The augmented sediment load caused by land degradation severely threatens to reduce biological diversity, crop yields, and nutrient levels globally by eliminating topsoil rich in nutrients [3]. Similarly, Vagen et al. [4] noted that the deposition of sediment yield in watersheds decreases the reservoir capacity and harms productivity, which causes food insecurity. This accumulation transported from the upstream river system caused a loss of reservoir storage and impacted its sustainability. Chuenchum et al. [5] predicted sediment yield in the 2050s and highlighted that reservoir storage capacity is disturbed yearly by 0.5–1% of sediment.

Furthermore, they noted that reservoirs of most dams worldwide would lose 50% of their current volume, which is a significant factor. Similarly, Walling [6] found that dams constructed in Asia lost nearly 40% of total reservoir storage due to soil erosion. This factor has an impact on the long-term viability of water resource projects. Recent studies show that developing countries such as India, Iran, Pakistan, and others suffer from increased sediment yield from major rivers in the watersheds. Nearly 20 billion tonnes (80%) of sediment n from the world's major rivers reach the oceans yearly [7]. This has severe threats to food production and the economy of the world.

The hydrological fluctuations in water and sediment inflow affect the storage volume of the reservoir and dam safety [8]. High suspended loads severely impact storage capacity and hydropower energy production by clogging bottom outlets and causing turbine abrasion [9,10]. Another major challenge associated with sediment deposition on hydropower systems is reducing the effective hydraulic width of flood protection. Furthermore, due to the remobilization of sediments from the reservoir, other technical issues in the downstream agricultural area have arisen due to sediment depositions [8]. Soil erosion and sedimentation, for example, cause land degradation, loss of livelihoods, canal siltation, and reservoir siltation in the upper Blue Nile basin [1,11]. Sediment production is strongly affected due to watershed characteristics, topography, geology, land use land cover, and rainfall seasonality, and it will have a significant global impact in the future [12,13].

Developing countries, including Ethiopia, spent a lot of money building hydropower plants to generate electricity. However, the storage volume of the reservoir and the efficiency of hydropower are decreasing due to the rapid increase in sediment in the watersheds. Some previous studies indicate that sediment yield temporal and spatial variation is a severe problem that censoriously alarms agricultural areas and hydropower sustainability. For example, siltation and nutrient enrichment levels significantly impact the reservoir of Glgel Gibe-I, as Devi et al. [14] investigated in the reservoir cross-section. Similarly, Tamene et al. [15] reported that the other hydropower project built downstream of Gilgel Gibe-I and II in southwestern Ethiopia, with a capacity of 1870 MW, is affected by similar effects. Within 23 years, approximately 3.5 million m^3,^ or 2300 t km^2^ of silt, has accumulated in the Koka reservoir [16]. The rapid increase in sediment load has also become a significant problem in some lakes, including Hawassa, Abaya, and Langano [[17], [18], [19]]. Hence, accurate silt deposition/sediment estimation and knowledge of sediment distribution in the area with the mitigation measures can play a significant role in the sustainability of the existing hydropower reservoirs and newly planned water resources projects. Besides, Jaiyeola and Bwapwa [20] stated that accurately estimating sediment yield conveyed from the watershed is needed to design various structures, such as erosion control structures, conduit pipes, and turbine blades.

In some cases, even small basins can contribute a maximum amount of sediment yield; thus, effective sediment yield reduction programs in the watershed are necessary. This requires estimating its quantity in the sub-basin and the area affected by highly vulnerable sediment accumulated [21]. According to Jansson [22], only 8.8% of the total watershed area contributes 69.1% of the sediment load. Moreover, the study of Lopez-Tarazon et al. [23] and Elosegi et al. [24] noted that quantifying deposit in highly erodible watersheds is essential to simulate potential effects on downstream reservoirs and select the best management practices.

In addition to controlling human activities in the watershed, it is necessary to consider sediment load in the basin or transported by river channel while planning and designing water resource projects. Adopting the management practices is another effective way to minimize the sediment yield entering the reservoir, which causes socioeconomic and maintenance costs in hydropower plants [25,26]. However, Ebabu et al. [27] noted that knowledge of sediment variations in both temporal and spatial terms at the watershed scale is essential before designing management options, particularly in severely impacted areas. Various BMPs adopted in different watersheds by different researchers and noted as effective in reducing erosion threats by trapping, contour tillage, slope tillage, afforestation, terrace farming, no-tillage, soil and water conservation measures, and cover crops have been noted as effective in reducing erosion threats in recent years. Bombino et al. [28] found that no-tillage is an efficient BMP that can reduce sediment yield by 75–80% in steep slope and clay soil areas in the Southern Calabria, Italy watershed. Similarly, Chen et al. [29] investigated the effectiveness of three management strategies for controlling sediment yield and observed that contour tillage, slope tillage, and conventional tillage can diminish soil erosion by 59.33–98.45% in red soil slope farmland. According to a review conducted by Desta et al. [30] on the land cover types on soil loss and crop productivity in Ethiopia, different land management practices: agronomic techniques, annual cropland cover, perennial vegetation coverage, drainage techniques, and area closure reduced sediment yield by 25–26.5%. Du et al. [31] discovered that no-tillage, cover crops, and agroforestry reduced soil loss by up to 80%. Terracing, contouring, and filter strips are commonly used in Ethiopian watersheds because they are more effective than others at reducing sediment yield. They can reduce sediment yield by up to 90% and are relatively inexpensive to implement and maintain. Overall, the identified BMPs reduced sediment loads with varying magnitudes at different watershed scales. As a result, various distributed models, such as the Water Erosion Prediction Project (WEPP) [32], SWAT [33], Limburg soil erosion model (LISEM) [34], European soil erosion model (EUROSEM) [35], agricultural non-point source pollution model (AGNPS) [36], and area non-point source watershed environmental response simulation (ANSWERS) [37], have been used in recent years to simulate the effectiveness of BMPs. For instance, according to the studies of Setegn et al. [38], Ayana et al. [39], and Sok et al. [40], SWAT has been successfully used to predict sedimentation yield in watersheds. SWAT is discovered by Dibaba et al. [41], Hassan et al. [42], and Leta et al. [43] to be capable of estimating sediment yield, identifying hotspot locations, and evaluating conservation strategies in Ethiopian watersheds under different scenarios.

Furthermore, Kefay et al. [44] confirmed that the SWAT could quantify sediment with its spatial distribution as well as identify hotspot areas for prioritization management approaches in Ethiopia. However, without hydrological models, it is difficult to quantify the intricate interactions between hydrological variables and evaluate the anthropogenic impacts on changes in sediment. Therefore, the SWAT model was used in this study to predict sediment yield and identify sediment yield hotspot subbasins for prioritization of best management practises. Three scenarios: filter strip, terracing, and contouring were considered to compare the effects of BMPs on Upper Gilo watershed. In addition, the spatial variability of erosion potential/sediment yield rate in the study area was compared based on the average annual sediment yield (tone/ha/yr) from each sub-basin to identify the area highly affected by sediment load with its management methods. The study's objectives were to (i) quantify the total sediment yield in the watershed, (ii) assess the spatiotemporal variability of soil erosion/deposits in the 45 sub-basins and identify the affected sub-basins due to high sediment rate, and (iii) compare the effectiveness of filter strip, terracing, and contouring in sediment reduction.

## 2 Materials and methods

### 2.1 Description of the study area

**Fig. 1:**
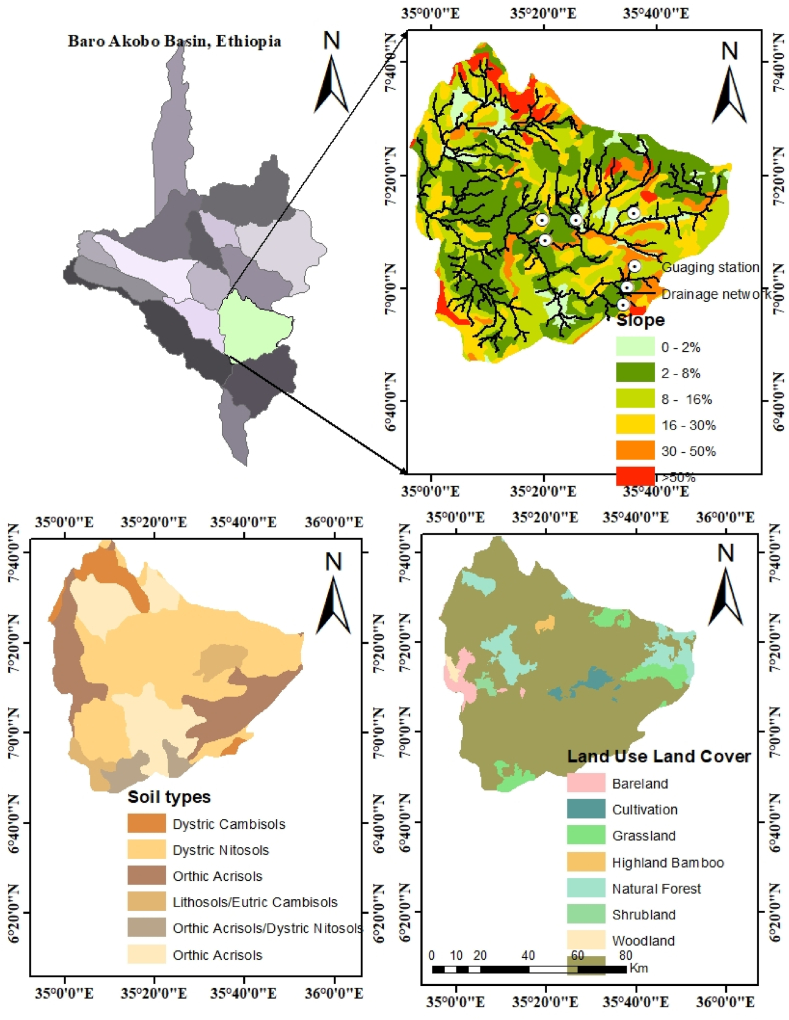
Location of the study area, soil type, and land use.

The upper Gilo watershed is located in southwest Ethiopia. Fig. 1 depicts slope, soil, and land use land cover of study area. The watershed is located between 6° 40′ 0″ and 7° 40′ 0″N latitude and 35° 0′ 0″ to 35° 40′ 0″E longitude, with elevations ranging from 508 to 2753 m above mean sea level. According to the slope map shown in Fig. 1, the mean slope ranges from 0 to 50%, with most of the area covered by moderate and gentle slopes covering 41.19% and 22.7%, respectively. The hydrological watershed area of the study area is approximately 6653.5 km^2^. As a result, the study area is one of the largest sub-basins in the Baro Akobo Basin. This study area touches three zones of western Ethiopia (Bench Maji Zone, Keffa Zone, and Shaka Zone) and two zones of Gambela (Majiang Zone and Agnuak Zone) and drains 111.2 km up to the basin outlet. In the study area, gravel roads connected southwestern Ethiopia and Gambela regions. However, the main asphalt road connecting Oromia (Jimma town) and Bonga to Guraferda crosses into the study area. High rainfall was distributed throughout the watershed in July and August.

### 2.2 Datasets used

Table 1 presents all of the data types used in this study. The Ethiopia Meteorological Agency (EMA) provided daily rainfall data for six stations (Aman, Mizan, Tepi, Benchi, Yeki, and Kura) from 1987 to 2020. The EMA also provided weather data like wind speed, sunshine, relative humidity, and solar radiation. The stations were selected within and around the upper Gilo watershed based on the data quality and recorded data periods. Because the normal annual rainfall of any neighboring exceeds 10%, the normal ratio method was used to fill in the missed rainfall data due to human and material errors. An inconsistency can occur due to instrument failure and observer error; therefore, the consistency was adjusted using the double mass curve method [45].

**Table 1:** Types of data used and their source.

Category of data	Description of data	Resolution (spatial/temporal)	Source	Period
Hydrological data	Sediment and runoff data that used for the calibration process.	daily	MWEE	1987–2020
Meteorological data	Daily precipitation, max/min temperature, humidity, wind speed, sunshine, and solar radiation of six stations.	daily	EMA	1987–2020
Digital elevation model	Alaska satellite facility DEM is used for watershed delineation.	12.5 m × 12.5 m	https://asf.alaska.edu	2020
Soil	Soil characteristics used to define HRUs, six soil types	30 m × 30 m	MoA	2013
Land use data	Seven land use classes used for defining of HRUs.	30 m × 30 m	EGSA	2020

*MWEE is the Ministry of Water and Energy of Ethiopia, MoA is the Ministry of Agriculture, EGSA is Ethiopian Geospatial Agency, and *DEM is the digital elevation model*.

A free 12.5 m × 12.5 m resolution digital elevation model was downloaded from the Alaska Satellite Facility website (https://asf.alaska.edu) and used to delineate the watershed and analyze terrain parameters such as slope, slope length, and stream network for each sub-basin. The slope map was reclassified into five categories: 0–5%, 5–8%, 8–15%, 16–30%, and greater than 30%. The soil data was obtained from the Ethiopia Ministry of Agriculture. The spatial distribution of six major soil types in the study area is shown in Fig. 1 and Table 2. The model spatial datasets were projected using ArcGIS 10.3, and the distribution of LULC, slope, and soil over the Gilo watershed is shown in Table 2. The Tepi gauge station, located at 35° 43′ 0″ E longitude and 7° 02′ 0″N latitude at the watershed outlet, has daily flow and sediment load datasets from 1987 to 2020.

**Table 2:** Summary of LULC, slope, and soil distribution over the study area.

S/No	Distribution of spatial data (LULC/slope/soil)		Area (km^2^)	Percentage of watershed area
1	Slope	0–5	2674.45	40.19
		5–8	1454	21.85
		8–15	1510.5	22.7
		16–30	952.9	14.32
		>30	61.7	0.93
2	Soil	Dystric nitisols	4719.8	71.1
		Orthic acrisols	213.2	3.2
		Calcic cambisols	19.8	0.3
		Dystric cambisols	23.2	0.3
		Calcic fluvisols	9.7	0.1
		Orthic solonchaks	948.6	14.3
		Leptosols	56.0	0.8
		Dystric fluvisols	55.6	0.8
		Calcic xerosols	118.5	1.8
		Eutric cambisols	388.8	5.9
		Dystric gleysols	32.5	0.5
		Eutric fluvisols	12.4	0.2
		Eutric nitosols	16.6	0.2
		Chromic luvisols	38.9	0.6
		Leptosols	4719.8	71.1
		Dystric fluvisols	213.2	3.2
		Calcic xerosols	19.8	0.3
3	LULC	Forest	2228.7	33.4
		Agricultural	923.5	13.8
		Wooded grassland	800.8	12.0
		Bushland	2718.3	40.7

### 2.3 SWAT model

**Fig. 2:**
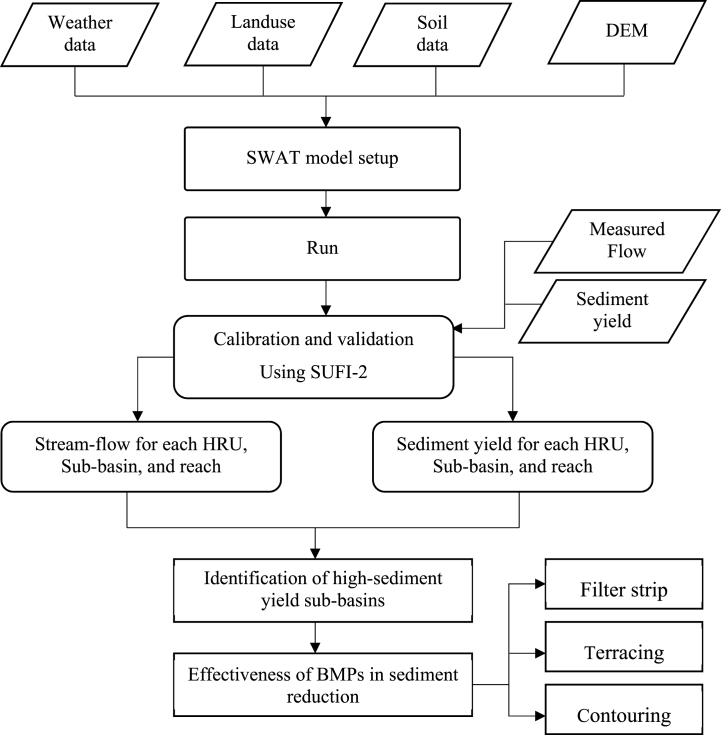
Flow chart showing overall methods used for simulation of sediment yield and effectiveness of BMPs.

The SWAT is a conceptual and variable time series model developed by the USARS (United States Agricultural Research Service) that is used to simulate hydrological responses, the rate of sediment yield, and nutrient loss at the watershed scale [46]. It is also a physically-based model that can quantify the impact of BMPs on water, sediment, and nutrient loss over a long period in large and complex watersheds with varying land use land covers, slopes, and soils [47]. SWAT was used in this study, because of it is widely used in large watersheds of Ethiopia. Based on watershed characteristics such as slope, land use land cover, and soils, model delineated the study area and divided into 45 sub-basins and several hydrological response units (HRUs). ArcSWAT uses the water balance equation to predict the hydrology at each HRUs. The overall methods used in this study was summarized in Fig. 2.

Land use, land cover, and DEM data are inputted into the SWAT model as a raster map, which describes the land cover type and elevation of each grid cell in the watershed. Soil data is inputted as a table that defines the soil properties for each grid cell in the watershed. This data can be obtained from soil map of the study area. SWAT then defines each land cover type with a four-letter code and each soil type with a three-letter code. The model can also used a weather generator for entering meteorological data. SWAT is a watershed-scale model that simulates runoff, agricultural chemicals, and sediment yields; it can also be integrated with the Modified Universal Soil Loss Equation (MUSLE) to predict the quantity of soil eroded from each hydrological response unit (HRU) in the watershed. The amount of sediment yield from each HRU is then summed to give the total soil erosion for the watershed. The MUSLE equation is a physically based equation, which means it depends on the physical processes that result in soil erosion. Furthermore, it is a more accurate model than the empirical equation (USLE) based on statistical relationships. The equation is used to deter-mine the quantity of sediment yield in SWAT by taking runoff, HRU area, soil erodibility, slope length and steepness, and coarse fragment cover into account. SWAT is integrated with MUSLE in this study to estimate more accurate soil erosion, examine the effects of land use and man-agement practices on sediment results, and evaluate the efficacy of the developed soil conservation strategies. The MUSLE equation in SWAT is as follows [48].

(1) $$\text{Sediment}\;\text{yield}=11.8\underset{0.56}{\left(\overset{\text{peak}}{q}x\overset{\text{Surf}}{Q}x\overset{\text{HRU}}{A}\right)}x\overset{\text{USLE}}{K}x\overset{\text{USLE}}{\text{LS}}x\overset{\text{USLE}}{P}x\overset{\text{USLE}}{C}\text{xCFRG}$$

Where; qp is the peak runoff (m^3^/s), QSurf is the daily runoff volume (mm), A_HRU_ is Area of hydrological response unit, K_USLE_ is the soil loss rate per erosion index unit (erodibility factor), L is the slope length, S_USLE_ is steepness factor, and C_USLE_ and P_USL_E are the soil loss ratios caused by land use and support practice, respectively. SWAT also integrated with the SCS curve number method to calculate the amount of surface runoff from each HRU in the watershed based on soil type, LULC, and antecedent moisture conditions. Eq. (2) shows the equation for estimating sur-face runoff (mm/ha) in SWAT using the SCS CN method. Rday is the rainfall depth for the day (mm), Ia is the initial abstractions (mm), S is the retention parameter (mm), as shown in Eq. (3), and CN is the curve number for the day.

(2) $$\overset{surf}{Q}=\frac{\underset{2}{\left(\overset{day}{R}-\overset{a}{I}\right)}}{\left(\overset{day}{R}-\overset{a}{I}+S\right)}$$

(3) $$S=25.4x\left(\frac{1000}{CN}-10\right)$$

The coarse fragment factor (CFRG) was computed using Eq. (4) based on the percentage of rock in the first layer:

(4) CRFG=exp(−0.053xrock)

### 2.4 Calibration and validation

The ArcSWAT model was calibrated and validated using monthly sediment yield and flow data from the Beko gauging station over 25 years. The model was calibrated and validated using data from 1990 to 2004 and 2005 to 2014. However, continuous flow and sediment data for 1987 to 1989 and 2015 to 2020 are only available for the rainy season and thus not considered for simulation in the present study. A large number of model parameters were estimated from various sources, influencing simulation results with varying weights [49]. As a result, SWAT-CUP was used before calibration to identify the sensitive parameters using a local sensitive approach (one parameter at a time). The SWAT-CUP is linked with the Sequential Uncertainty Fitting method (SUFI-2) to per-form sensitivity analysis and rank the inputted parameters based on their order of sensitivity by comparing observed and simulated results [50]. SUFI 2 is a global optimization algorithm for identifying uncertainty in parameters, then calibrating and evaluating the uncertainty of watershed models. It is simple to use and can handle a large number of parameters. According to Khalid et al. [51], uncertainty due to conceptual model, parameter estimation, driving variable, and observed data are all taken into ac-count in SUFI-2. Numerous studies e.g. Ayele et al. [52], Azari et al. [53], and Yesuf et al. [50] concluded that SUFI is a powerful and time-saving sediment yield calibration tool. In SWAT-CUP, the parameters influencing runoff were calibrated first, followed by the parameters influencing sediment yield. For example; runoff curve number (CN2) is an important parameter in estimating runoff and sediment yield. It is a dimensionless number that is used to represent the infiltration capacity of the soil. The lower the CN2, the more permeable the soil is and the less runoff generated; this means that the runoff transports less sediment. Furthermore, the Manning roughness coefficient value for the main channel (CH–N2) and effective hydraulic conductivity in the main channel (CK_K2) are estimated based on channel characteristics such as bed material, vegetation, and sediment quantity. The lower the CH_N2, the smoother the channel is and the less resistance there is to flow. This means that the flow velocity will be higher and more sediment will be transported. Conversely, the higher the CH_N2, the rougher the channel is and the more resistance there is to flow. This means that the flow velocity will be lower and less sediment will be transported. The CH_K2 is an important factor in estimating sediment yield because it can be used to estimate the flow velocity in the channel. The higher the CH_K2, the easier it is for water to flow through the channel. This means that the flow velocity will be higher and more sediment will be transported. SWAT parameters are classified into six groups: basin, sub-basin, main channel, HRU, groundwater, and soil. To determine the sensitivity of a parameter, two statistical measurements (P-value and t-value) are used. The sensitivity range is represented by the t-value, but the significance of sensitivity is represented by the P-value.

We evaluated SWAT model performance by comparing observed and simulated outputs using Nash-Sutcliffe modeling efficiency (NSE), coefficient of determination (R^2^), and percent bias (PBIAS) [[54], [55], [56], [57], [58]]. The NSE (Table 3) is calculated as a ratio of simulated to observed variances, ranging from − ∞ to +1. While NSE values less than 0.5 indicate an unsatisfactory model, values greater than 0.75 imply that model simulations are very good [59]. The PBIAS indicated whether the average simulated results were greater or lesser than the observed data, and for sediment ranges from -∞ to +∞. If the value is negative, the simulated results are overestimated; if it is positive, the results are underestimated [60]. The R^2^ value ranges from 0 to 1, representing the relationship between the simulated and observed data. If the R2 value is one, the simulated and observed data are equal. If R2 is zero, it indicates no correlation between the simulated and observed data [61].

**Table 3:** Statistical efficiency criterion, statistic equation, value range, and model performance ratings for sediment yield.

Statistical efficiency criterion equation	Range	Performance classification	References
$NSE=1-\left[\frac{\sum_{i=1}^{n}\underset{2}{\left(\underset{obs}{Yi}-\underset{sim}{Yi}\right)}}{\sum_{i=1}^{n}\underset{2}{\left(\underset{obs}{Y}-\underset{obs}{\bar{Y}}\right)}}\right]$	0.75<ESN≤1 0.65<ESN≤0.75 0.5<ESN≤0.65 ESN≤0.5	Very good Good Satisfactory Unsatisfactory	[62]; [1]
$\underset{2}{R}=\frac{\underset{2}{\left[\left(\overset{obs,i-\overset{obs}{\bar{Y}}}{Y}\right)\left(\overset{sim,i-\overset{sim}{\bar{Y}}}{Y}\right)\right]}}{\sum_{i=1}^{n}\underset{2}{\left(\overset{obs,i-\overset{obs}{\bar{Y}}}{Y}\right)}\sum_{i=1}^{n}\underset{2}{\left(\overset{sim,i-\overset{sim}{\bar{Y}}}{Y}\right)}}$	R^2^ < 0.5 0.5<R^2^ < 0.6 0.6<R^2^ < 0.7 0.7<R^2^ < 1	Unsatisfactory Satisfactory Good Very good	[47]
$PBIAS=\frac{\sum_{i=1}^{n}\left(\underset{obs}{Yi}-\underset{sim}{Yi}\right)\;x\;100}{\sum_{i=1}^{n}\underset{obs}{Yi}}$	PBIAS≥±55 ±30≤PBIAS<±55 ±15≤PBIAS<±30 PBIAS<±15	Unsatisfactory Satisfactory Good Very good	[47,59].

*n is the number of observations, Yi^obs^ represents observed variables, Yi^obs^ is simulated variables, $\overset{\text{sim}}{\bar{Y}}$ and $\overset{\text{obs}}{\bar{Y}}$ are the mean of simulated and observed values in respective time steps i.

### 2.5 Sediment yield reduction scenarios

Sediment yield varies with topography, vegetation cover, rainfall seasonality, and soil disturbance as a result of land use land cover, gradually reducing reservoir storage capacity. It also threatens agricultural land and water resources; thus, estimating sediment yield and identifying hotspot areas within the watershed is essential for watershed management. In addition to identifying the spatial variation of sediment, this study identified the temporal variability of sediment yield by estimating the average monthly sediment yield. We used the widely used effective BMPs to reduce sediment yield. Different BMPs, such as contour tillage, strip tillage, terraces, fertilizer application changes, annual cropland cover, perennial vegetation coverage, crop rotation, reforestation, and so on, are effective in sediment control at different watershed scales, as recommended in various studies [46,[63], [64], [65]].

Three BMPs were simulated in the SWAT model based on the available field (i.e. the area of land available for agricultural use and the area defined by other land use criteria such as land cover, slope, and other factors) and existing agricultural practises to evaluate the management strategy efficiency. The SWAT was simulated in each of the following scenarios: baseline, filter strip, terracing, and contouring. Scenario So (baseline or current condition) was used to simulate the sediment yield without management options. The calibrated parameters and other values suggested by the literature were used as input parameters for the baseline situation. The baseline scenario's simulated result is used as a reference to compare the reduction capacity of selected BMPs. However, as a result of human activity, the watershed's sediment yield is increasing over time. As a result, the BMPs were represented in calibrated models in scenarios 1 (filter strip), 2 (terracing), and 3 (contouring) by varying the input parameters in order to simulate the effects of each on the SWAT simulation response. In Scenario 1, filter strips were implemented on crops, dry land, soil types, pasture, and slope classes. This BMP helps to slow runoff, reduce soil erosion, improve infiltration, and improve sediment tapping. We adjusted the width of filter strips from 1 m to 30 m based on local study experiences in Ethiopia's highlands to evalaute the influence of filter strips on sediment trapping [15,66]. Two other structural best management practices were developed in scenarios 2 and 3 to evaluate the impact of terracing and contouring on sediment control, respectively. Contouring is a type of tillage that helps mitigate soil erosion by slowing surface runoff and allowing it to penetrate by impounding it [41]. This scenario assessed contouring in agricultural lands by modifying the soil conservation service curve number (CN2) and USLE Practice factor (USLE_P) in SWAT based on the land slope, literature reports, and SWAT user manual recommendations [44].

In scenario 3, terracing is assigned to reduce soil erosion by constructing specific ridged platforms (terraces) in a hillside. Earlier studies, such as Woznicki and Nejadhashemi [67] and Dibaba et al. [41], suggested terracing combined with contour farming is more effective than other mitigation practices. Terracing controls sediment yield because it allows for more intensive cropping systems to be used by avoiding the formation of gullies and allowing sediment from surface runoff to settle. The effectiveness of each BMP scenario was determined by altering the model parameter values. Eq. (5) is used to estimate sediment reduction.

(5) $$\text{Sediment}\;\text{reduction}\;\left(\%\right)=\left[\left(\text{Sed}.Y_{1}-\text{Sed}.Y_{2}\right)/\text{Sed}.Y_{1}\right]x\;100$$

Where; Sed.Y_1_ is the sediment yield before BMP implementation, and Sed.Y_2_ is the estimated sediment after BMP implementation.

## 3 Result and discussion

### 3.1 Sensitivity analysis, calibration, and validation

A sensitivity analysis identified the parameters that had a significant effect on the flow and sediment yield. SUFI2 automatically identified the preferred ranges. In addition, the parameters were ranked based on their influence on the hydrological model simulation results and the P-value and t-stat. The results show that the most sensitive parameters for flow simulations are CN2 (runoff curve number), CH_N2 (Manning's "n" value for the main channel), CH_K2 (Effective hydraulic conductivity in the main channel), and ALPHA_BF (Base flow alpha factor), ranked 1 to 4. The sensitive parameters, recommended range, and fitted values are shown in Table 4, Table 5. To reduce uncertainty in the model result, the flow and sediment yield parameters have been adjusted within their ranges. According to the findings, parameters with a higher t-stat and a lower p-value have a significant impact on model results in the study area. Similarly, Betri et al. [66] and Himanshu et al. [59] found CN2, CH_K2, ALPHA_BF, and RCHRG.DP are the most sensitive to flow prediction. However, in Abebe and Tolessa [57], Sol_AWC (available water capacity), Sol_Z (soil depth), and Sol_K (Soil conductivity) are the most sensitive flow parameters ranked 1 to 3. The degree of sensitivity of parameters varies from area to area because the parameters can be affected by the physical characteristics of the sub-basin. For example, according to Abebe and Gebremariam [68], the sensitivity of available water capacity (Sol_AWC), soil depth (Sol_Z), and Plant evaporation compensation factor (Epco) are high to affect the flow simulation value in Kesem dam watershed. Moreover, groundwater delay time, shallow aquifer water depth threshold for revap, and soil evaporation compensation factor are low-sensitive parameters affecting the flow of the Gilo watershed.

**Table 4:** Most sensitive parameters of flow in the Upper Gilo watershed.

Parameter code	Description	Ranges	Fitted value	Sensitivity order
CN2.mgt	Runoff curve number of SCS	±20%	−0.16%	3
CH_N2.rte	Manning's "n" value for the main channel	0.01 to 0.3	0.21	1
GWQMN.gw	Threshold depth of water in the shallow aquifer (mm)	−1000 to +1000	−723.6	8
RECHARGE.gw	Percolation fraction of deep aquifer	0–1	0.87	5
SOL_AWC.sol	Available water capacity of the soil layer (mm H2O/mm soil)	±20%	−1.86%	9
GW_DELAY.gw	Groundwater delay time (days)	0 to 470	45.8	11
CH_K2.rte	Effective hydraulic conductivity in the main channel (mm/hr)	100 to 500	368.2	2
REVAPMN.gw	Threshold water depth in the shallow aquifer for revap	−100 to +300	−131.5	12
SOL_K.sol	Saturated hydraulic conductivity(mm/hour)	±20%	0.98333	6
EPCO.bsn	Soil evaporation compensation factor	0–1	0.965	7
GW_REVAP.gw	Groundwater revap co-efficient	0.02–0.2	0.185	10
ALPHA_BF.gw	Base flow alpha factor	0.8 to 1	0.85	4
ESCO.hru	Soil evaporation compensation factor	0–1	0.916	13

*mgt is crop cover management, rte is water routing, gw is groundwater, bsn is the entire watershed scale, sol is soil water dynamics, and hru is water dynamics at the HRU level.

**Table 5:** Most sensitive parameters of sediment yield in the Upper Gilo watershed.

Parameter	Description	Range	P_value	t_value	Sensitivity rank
SPCON	Parameter for channel sediment routing	0.0001–0.01	0.181	−0.577	6
CH_COV2	Channel cover factor	0.001–1	0.583	−0.558	7
USLE_K	USLE equation soil erodibility factor	0–0.1	0.018	1.386	5
USLE_P	USLE equations support practice factor	0–1	0.563	1.431	4
CH_COV1	Channel erodibility	0.01–0.6	0.721	1.899	3
HRU_SLP	Average slope steepness	0–5000	0	−13.446	1
SLSUBBSN	Average slope length	±20%	0.022	2.487	2
SPEXP	Exponential parameter	1–2	0.98	−0.00253	9
RSDIN	Initials residue factor	0–1000	0.789	−0.271	8

**Fig. 3:**
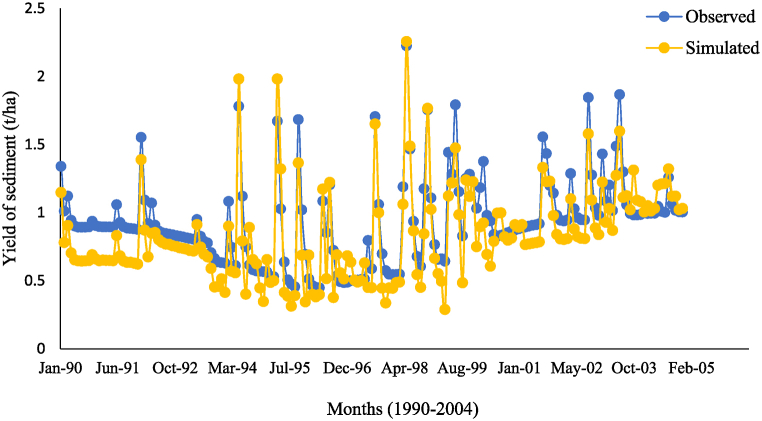
The observed and simulated sediment yield of the Beko station during calibration.

**Fig. 4:**
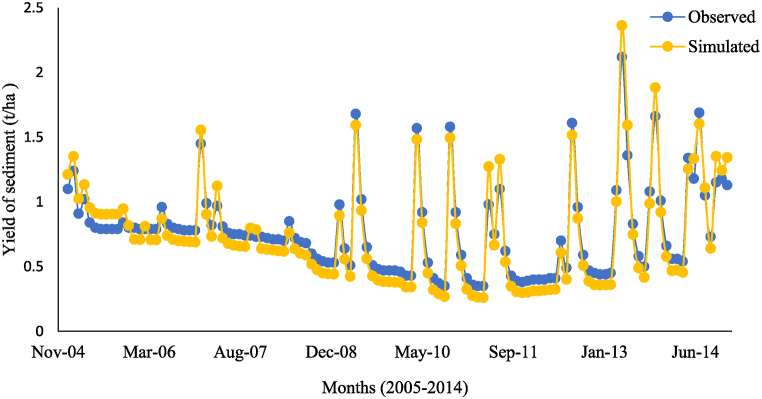
The observed and simulated sediment yield of the Beko station during validation.

In addition, the model is calibrated for sediment after the SWAT has been calibrated and validated for flow. The amount of sediment in the watershed is determined by the complex relationship of terrain, land use, vegetation, climate, and soil. The results of the sensitivity analysis of sediment yield indicated that nine parameters were the sensistive parameters affects the sediment simulation, the result of *t*-test and P-values are presented in Table 5. Average slope steepness (HRU_SLP), Average slope length (SLSUBBSN), and Channel erodibility (CH_COV1) are very sensitive influencing the sediment yield in the upper Gilo watershed and were ranked 1 to 3. These parameters are used to determine the sediment concentration from the watershed. Those flow and sediment parameters were adjusted from the model's initial estimates to fit the SWAT simulations with the observed data. Fig. 3, Fig. 4 show good agreement between simulated and observed sediment yield hydrographs for monthly time steps at the Beko gauging station during the calibration and validation period.

The simulated matched the observed monthly flow for the calibration with values of NSE, PBIAS, and R^2^ equal to 0.69, −12.6%, and 0.71, respectively. For the validation, the observed and simulated flow showed acceptable agreement as indicated by NSE, PBIAS, and R^2^ equal to 0.59, −6.4%, and 0.68, respectively. Likewise, the NSE, R^2^, and PBIAS for sediment yield calibration of the Beko gauging station were 0.64, 0.69, and −10.3%, respectively, and that for validation were 0.53, 0.61, and −3.4%, respectively. These evaluation statistics show that the model performance is acceptable for the upper Gilo watershed in simulating flow rate and sediment concentration. This demonstrates that the SWAT has an exceptional capacity to simulate both flow and sediment yield with the effectiveness of management practises [46].

### 3.2 Sediment yield variation in the watershed

It is crucial for watershed management to estimate the variation of sediment yield in the spatiotemporal. The results show that flow from the watershed is extremely high during the rainy season, with a high sediment yield of 1021.8 tons/ha/yr. In addition, the monthly average sediment yield is 3.76 tons/ha. On the other hand, sediment concentrations in each sub-basin are not uniform. This is consistent with earlier study findings. For example, Negewo and Sarma [21] and Das et al. [69] noted that the amount of sediment within the watershed is highly variable over space and time. Even in the small portion of the area that is not uniformly contributed due to land use, slope, and soil types. It is crucial to determine the hotspots area (the area has high sediment yield) in order to prioritise mitigation measures and track their effectiveness. Governments and other organizations can help reduce sediment and safeguard their water resources, wildlife, and property by identifying and targeting hotspots.

**Fig. 5:**
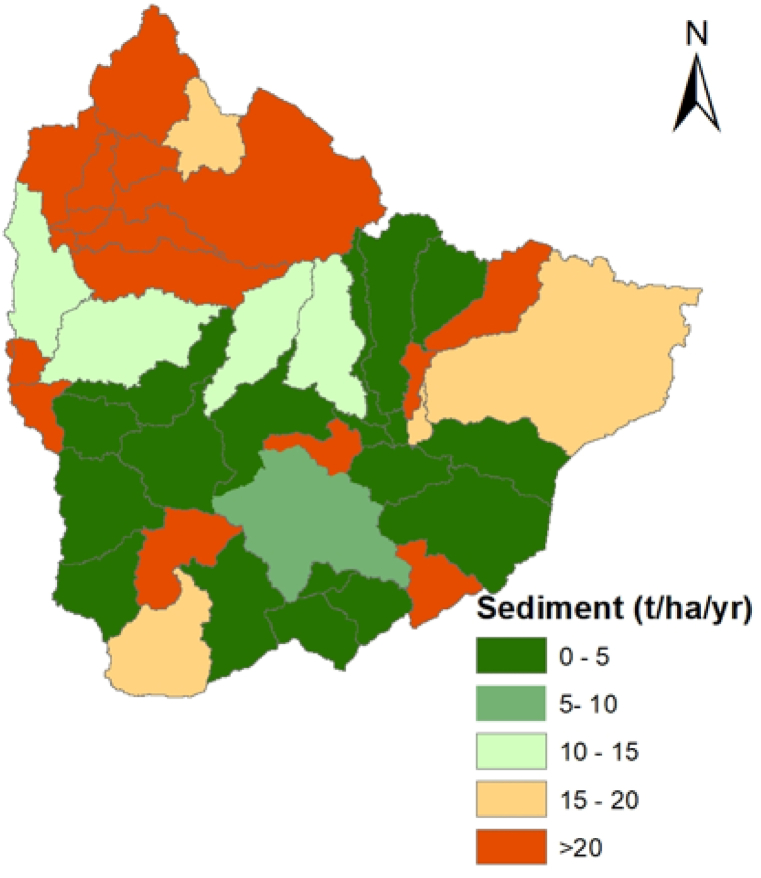
Sediment Yield map of Upper Gilo Watershed.

Sub-basins 14, 37, and 40 have the highest sediment yield, with annual average values greater than 41 tons/ha/yr, according to Fig. 5. Natural processes like river/stream nature, geological condition, land use, topography, and rainfall seasonality all contribute to spatial variations in sediment yield per unit area. Deforestation, agriculture, overgrazing, and other forms of watershed disturbance are also substantial contributors to variation in sediment loads within the study area. Accordingly, it is beneficial to identify the most sediment-yielding sub-basins using a simulation of spatiotemporal variations to control the sediment from the watershed [70]. Table 6 and Fig. 5 shows sub-basin classification based on the average yearly sediment yield. According to the findings of this study, the annual mean sediment yields in six sub-basins 5, 10, 11, 15, 22, and 31 exceeded 20 tons/ha/year (i.e., very high); however, sediment yield in eighteen sub-basins are extremely low, less than 5 tons/ha/year. These yield type classification used to identify highly affected sub-basins within watersheds is based on previous research done in Ethiopian watersheds [41,57].

**Table 6:** Classification of sub-basins based on the average annual sediment yield.

S.No	Yield type	Sediment yield (tons/ha/year)	Sub-basins	Percentage (%)
1	Extremely high	>41	11 (1, 3, 4, 6, 7, 8, 9, 14, 17, 37 & 40)	24.44
2	Very high	21–40	6 (5, 10, 11, 15, 22, & 31)	13.33
3	High	16–20	4 (2, 25, 26, & 45)	8.89
4	Medium	11–15	1 (20)	2.22
5	Low	6–10	2 (38, 39)	4.44
6	Extremely low	0–5	18 (13, 18, 19, 23, 24, 27, 28, 29, 30, 32, 33, 34, 35, 36, 41, 42, & 43)	40

**Fig. 6:**
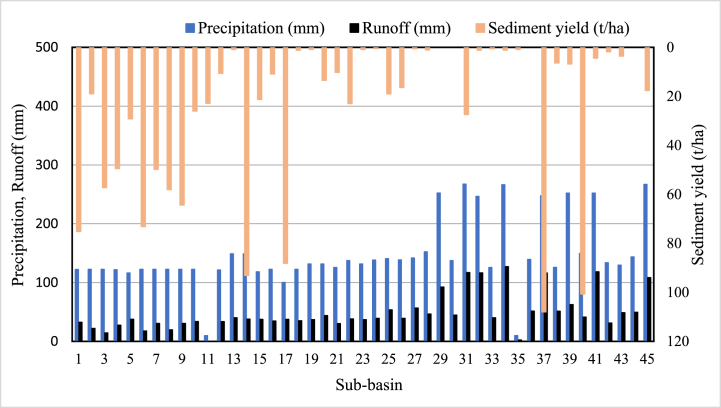
Comparison of precipitation, runoff, and sediment yield in the study area.

**Fig. 7:**
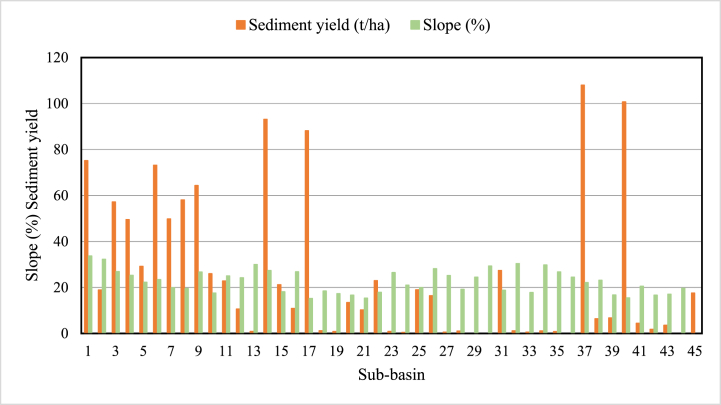
Comparison of sediment yield and slope of each sub-basin.

In the 24.44% of the study area, the sediment yield is extremely high (i.e., more than 41 tons/ha/yr). These areas are almost bare land and covered by open bushes and grass. However, 40% of the total area contributes to the minimum sediment concentration because the surface runoff from other sub-basins cannot pass through these areas and because they are mostly covered by forest. Consistent with these results, Martinez-Martinez et al. [71] noted that sediment yield varies spatially due to natural and human activities. Due to more frequent severe precipitation and runoff generation than in lower sub-basins, most upstream areas, such as sub-basins 1, 3, 4, and 6, contribute to extraordinarily high sediment yield. This indicated that the upper part of watershed requires land management to minimize the effects of sediment yield on the downstream area. In particular, the sub-basin 37 near the outlet has the highest sediment accumulation. This area has the maximum rainfall distribution (average monthly rainfall is 247.41 mm) and a more than 20% slope. Tadesse et al. [72], Abebe and Gebremariam [68], and Sadhwani et al. [73] have also previously highlighted the high sediment yield and its significant effects in sub-basins with high rainfall distribution and runoff. The amount of sediment that is carried by runoff depends on the amount of runoff, the type of soil, and the vegetation cover. In general, more runoff will result in more sediment being carried. This is because more runoff means that there is more water available to carry sediment. The simulation results of the spatial distribution shown that sub-basins 37 and 40 have high sediment yield and runoff among the 45 sub-basins of the Upper Gilo watershed. Sub-basin 37 (2968.9 mm runoff rates per year and 107.97 tons/ha) and 40 (1791.7 mm runoff rates per year and 100.7 tons/ha) are in the highest runoff rate and sediment yield distributions. However, as shown in Fig. 6, precipitation and runoff do not affect sediment yield; in some sub-basins, high rainfall and runoff contribute less sediment. For example, sub-basins 27, 28, 29, and 31 have high rainfall (greater than 130 mm average monthly rainfall) and contribute high runoff (greater than 44.63 mm average monthly runoff), but, the contributed sediment yield from these sub-basins very low (less than 1.03 ton/ha). This indicated that only precipitation and runoff have no effect on the sediment yield. As a result, other factors such as land use, soil, slope, and topography are considered when estimating sediment yield. The watershed slope is an important factor in determining sediment; steeper slopes have larger sediment yields than gentler slopes. In Fig. 7, the results of classified slope value and sediment yield of the sub-basin are compared to confirm this. However, always the relationship between slope and sediment yield is not linear because other factors such as soil type, vegetation cover, and rainfall can all affect the relationship between slope and sediment yield.

**Fig. 8:**
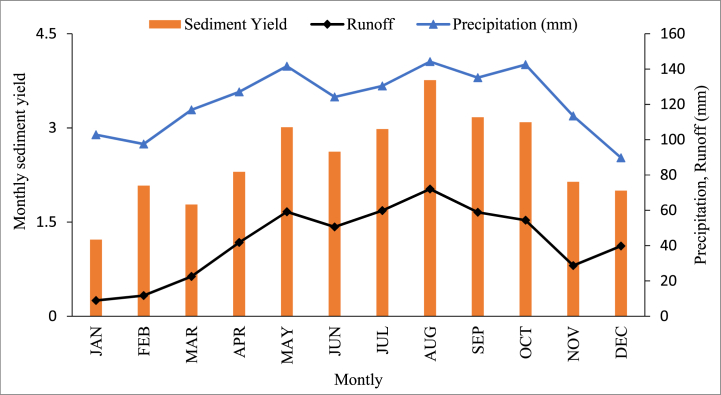
Comparison of mean monthly sediment yield, precipitation, and runoff.

According to Fig. 8, high monthly precipitation events contributed to a high sediment yield each year. The sediment concentrations are very high and low in August and January. Furthermore, runoff and sediment yield are more in rainy season months such as July, August, and September than in other months in the area of study. Thus, it can be concluded that sediment yield varies with season and directly correlates with runoff.

### 3.3 Effects of best management practices on sediment yield

In the study area, sub-basins contribute higher annual average sediment yield, necessitating particular management practises. The six (8.89%) and eleven sub-basins (24.44%) are classified as very high and extremely very high sediment yield areas because they contribute more than 20 tons/ha/year on average. High sediment can have a negative impacts on the environment by reducing water quality, eroding beaches and shorelines, clogging waterways, and causing infrastructure damage. Sediment transports pollutants like nutrients and pesticides, which difficult for aquatic life to grow, harm fish. and makes water unfit for drinking and recreation. In addition, it can also cause flooding, which can damage bridges and other infrastructures. According to experts and earlier studies suggestion, BMPs have to be considered to reduce sediment yield by protecting soil from erosion, reducing runoff, and capturing silt before it enters waterways [[41], [42], [43]]. After identifying hotspots in the watershed, BMPs will be implemented to reduce sediment yield and protect our water resources, wildlife, and property. Several factors such as the yield type, the cost of implementation, long-term sustainability, the efficiency of the BMPs in reducing sediment, the amount of rainfall, land use, and the slope of the sub-basins should be considered while selecting appropriate management practices. As a result, in this study, inexpensive and more effective BMPs for reducing sediment yield were applied to those sub-basins greater than 20 tons/ha/year [57].

**Fig. 9:**
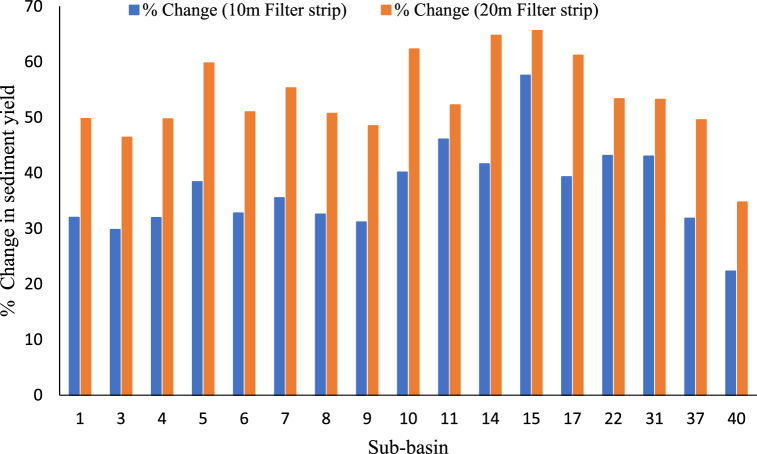
Effectiveness of Filter strip on the monthly sediment reduction.

The sediment yield from prone sub-basins (14, 17, 37, and 40) is reduced by 22.3–57.6% in scenario 1 due to the use of a 10 m wide filter strip (Fig. 9). According to simulation results, the upper Gilo watershed total sediment yield at the outlet is reduced from 966.5 tons/ha/year to 628.84 tons/ha/year (34.9% from the baseline). However, when the 20 m filter strip was used, the sediment yield reduction percentage increased from 34.7 to 65.6% (i.e., the total baseline sediment yield was reduced from 966.5 to 463.6 tons/ha/year). These findings show that increasing the width of the filter strip can increase the reduction efficiency of the sediment yield-prone sub-.basins. This study's results are comparable to previous studies conducted in different watersheds. For example, Risal and Parajuli [46] found that applying filter strips with 10, 20, and 30 m widths at the edge of agricultural fields in the Big Subflower River and Stovall Sherard watershed reduced sediment yield by 9%, 11%, and 12%, and 12%, 33%, 38% respectively. Similarly, Abebe and Tolessa [57] noted that increasing the width of filter strips from 5 m to 10 m reduced sediment yield by 43.58–70.11% and 62.3–80%, respectively, from Ethiopia's Kesem Dam Watershed.

**Fig. 10:**
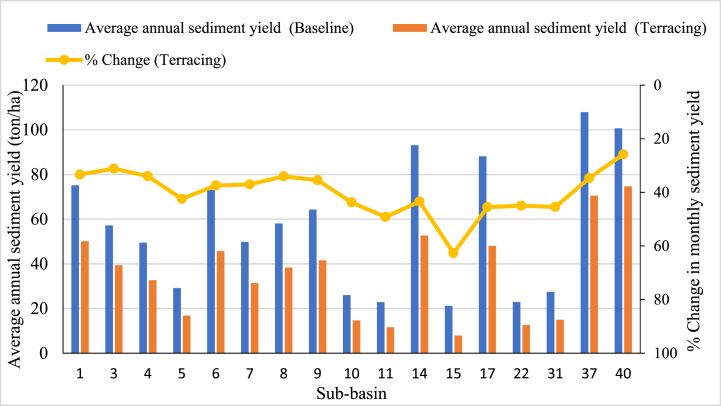
Effectiveness of terracing on the monthly sediment reduction.

Moreover, the sediment yield by series horizontal ridges, i.e., terraces constructed on hillsides and high rainfall sub-basins, reduced to 362.9 tons/year from the watershed, equivalent to a 37.5% reduction. Furthermore, terracing management options reduced sediment from those sediment-prone sub-basins by the highest percentage. This indicated that 25.8%–62.7% of sediment yield is reduced from high sediment-yielding sub-basins (Fig. 10). Hence, implementing terracing is another option for minimizing runoff and soil erosion and significantly reducing sediment yield [48,59,74]. Compared to all simulation scenarios, implementing an in-situ soil conservation method (contouring) would be more beneficial than implementing filter strips and terracing, as summarized in Table 7. This is consistent with Himanshu et al. [59], Abebe and Tolessa [57], and Kitheka et al. [75] study.

**Table 7:** Comparison of effectiveness of management practises for reducing sediment yield.

Scenarios	Baseline	Filter strip		Terracing	Contouring
		20 m	30 m		
Total sediment yield from prone sub-basins	966.5	628.8	463.6	603.6	578.2
% Reduction from the baseline scenario	–	34.9	52.0	37.5	40.2

**Fig. 11:**
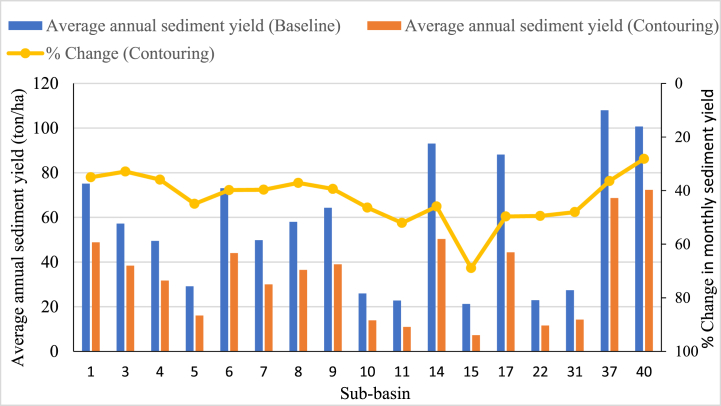
Effectiveness of contouring on the monthly sediment reduction.

The impact of best management practices (e.g., contour farm scenario) revealed a significant reduction ratio of up to 68.9% from all sub-basins, as shown in Fig. 11. This management strategy would ensure minimal soil displacement and loss of soil fertility in the watershed; however, filter strips would trap eroded sediments. Sub-basins 5, 6, 7, and 8 had the highest sediment yield under the contouring scenario, with percentage reductions of 68.1%, 52.1%, 49.7%, and 49.4% from the baseline scenario, respectively. Sub-basins 15, 11, 31, and 10 exhibited the relatively highest sediment yield due to terracing. While sub-basins 3, 9, 37, and 40 had the lowest sediment reduction under the filter strip scenario, and sub-basins 1, 3, 4, and 40 had the lowest sediment yield under the contouring scenario. These findings indicated that while implementing different management options has varying effectiveness, combining them would be more beneficial than applying individual management practices. Three BMPs reduced the sediment yield of vulnerable areas from high to low [76]. Thus, selecting sediment management practises is extremely beneficial for decision-makers, policymakers, water resource engineers, and hydro-ecologists [65]. Overall, the highest percentage of reductions was observed at the sub-basin level compared to the watershed level, consistent with previous studies' findings [74,77].

However,the efficiency of BMPs for sediment yield might vary depending on the specific conditions of the each sub-basin, how they are implemented, and climate change. As a result, public participation is critical for the successful implementation of BMPs; consequently, people must be informed of the importance of reducing sediment production and the ways that they can implement. An expert should assist people in increasing the efficiency of BMPs by selecting BMPs that are appropriate for specific watersheds, correctly implementing BMPs, monitoring BMP efficacy, and making adjustments to BMPs as needed. Furthermore, different levels of land managers and experts can play a vital role in increasing the efficiency of BMPs in a variety of ways, including providing technical advice and guidance to farmers on how to implement, modifying existing BMPs and developing new BMPs to make them more effective, and educating and training stakeholders such as farmers and policymakers about the benefits of BMPs and encouraging their adoption. Through public awareness campaigns, educational programmes, and other outreach efforts, the country's policy should encourage the use of BMPs, regulate land use, and educate the people about the consequences of sediment yield and the importance of implementing BMPs. Ethiopian governments, as well as different stakeholder groups, can help to mitigate the effects of sediment yield in the Upper Gilo Watershed and protect their water resources, wildlife, and property through future research and creating incentives. Furthermore, to track the effectiveness of mitigation solutions, it could be necessary to promote sustainable agricultural practises (such as terracing, contour farming, and cover cropping), reforestation of degraded land, improvement of drainage systems, and identification of hotspots.

## 4 Conclusion

This study used the SWAT to estimate sediment yield at sub-basins and watershed scales and evaluate the effectiveness of best management practices in sediment reduction in the Upper Gilo Watershed, Ethiopia. Statistical efficiencies such as NSE, R^2^, and PBIAS show SWAT acceptance in simulating runoff and sediment yield. Three different BMP scenarios were implemented in high-prone areas: filter strip, terracing, and contouring, and their impacts on sediment accumulation were evaluated at both the sub-basin and watershed scales. The study findings revealed that the annual sediment yield from all sub-basins is 1021.8 tons/ha/yr. Within the study area, the spatial distribution of annual sediment yield ranges from 0 to 107.97 tons/ha/yr. About 37.78% of the sub-basins contributed more sediment yield than the others (greater than 20 tons/ha/yr). In this study, eleven sub-basins, six sub-basins, two sub-basins, and the remaining eighteen sub-basins are highly affected areas, moderately affected, low erosion areas, and very low sediment prone areas, respectively. Similarly, the distribution of sediment yield varies within the year from month to month; the rate is extremely high in August because of heavy rainfall. BMPs scenario showed that each management option greatly affects sediment reduction at both watershed scale and sub-basins. Contouring is the most effective practice for reducing sediment up to 48% among each management option. Likewise, the filter strip has different efficiency during its width is increased from 10 m to 20 m. Up to 43% and 53.2% of sediment is reduced from prone sub-basins when 10 m and 20 m wide filter strips are implemented, respectively. However, terracing had little effect on sediment yield reduction after strip filters. Overall, the reduction potential of the selected BMPs is different; this caused due to the variation of DEM, LULC and soil types in the study area. The findings indicated that after identifying the variation of sediment yield in the sub-basins, identifying the most sediment-yielding sub-basins is very useful for proposing the best management strategies to reduce the sediment caused by different problems in the watershed. Other watershed decision-makers will use the finding of this study to manage the soil erosion by evaluating the effectiveness of BMPs for reducing sediment yield at the sub-basin and watershed levels. When planting crops, vegetation, slope, and available materials of tone, soil, or concrete are taken into account, all selected management options are effective and sustainable for mitigating the effects of sediment. However, this study is not addressed the costs, life cycle, and operation of management practices due to local constraints such as geographic challenges, economic expense, and resource availability. Therefore, further studies could analyze the uncertainty and fill this gap in the study area.

## Funding

This research did not receive any specific grant from funding agencies in the public, commercial, or not-for-profit sectors.

## Author contribution statement

Mengistu Zantet oybitet: Conceived and designed the experiments; Analyzed and interpreted the data; materials, analysis tools or data; Wrote the paper.

Takele Sambeto Bibi: Analyzed and interpreted the data; materials; Wrote the paper. Eliyas Abdulkerim Adem: Materials, analysis tools or data; Wrote the paper.

## Data availability statement

Data will be made available on request.

## Declaration of competing interest

The authors declare no conflicts of interest.
